# Adult Primary Spinal Epidural Extraosseous Ewing's Sarcoma: A Case Report and Review of the Literature

**DOI:** 10.1155/2016/1217428

**Published:** 2016-08-17

**Authors:** Mark Bustoros, Cheddhi Thomas, Joshua Frenster, Aram S. Modrek, N. Sumru Bayin, Matija Snuderl, Gerald Rosen, Peter B. Schiff, Dimitris G. Placantonakis

**Affiliations:** ^1^Department of Neurosurgery, NYU School of Medicine, New York, NY 10016, USA; ^2^Department of Pathology, NYU School of Medicine, New York, NY 10016, USA; ^3^Perlmutter Cancer Center, NYU Langone Medical Center, New York, NY 10016, USA; ^4^Brain Tumor Center, NYU Langone Medical Center, New York, NY 10016, USA; ^5^Department of Medicine, NYU School of Medicine, New York, NY 10016, USA; ^6^Department of Radiation Oncology, NYU School of Medicine, New York, NY 10016, USA; ^7^Kimmel Center for Stem Cell Biology, NYU School of Medicine, New York, NY 10016, USA

## Abstract

*Background*. Extraosseous Ewing's sarcoma in the spinal epidural space is a rare malignancy, especially in adults.* Case Presentation*. A 40-year-old male presented with back pain and urinary hesitancy. MRI revealed a thoracic extradural mass with no osseous involvement. He underwent surgery for gross total resection of the mass, which was diagnosed as Ewing's sarcoma. He was subsequently treated with chemoradiotherapy. He remains disease-free 1 year after surgery. Review of the literature indicated only 45 previously reported cases of spinal epidural extraosseous Ewing's sarcoma in adults.* Conclusions*. Extraosseous Ewing's sarcoma in the spinal epidural space is a rare clinical entity that should be included in the differential for spinal epidural masses. Its treatment is multidisciplinary but frequently requires surgical intervention due to compressive neurologic symptoms. Gross total resection appears to correlate with improved outcomes.

## 1. Introduction

Ewing's sarcoma (ES) is a malignant bone tumor of childhood and adolescence that occurs primarily in the diaphysis of long bones, such as femur, tibia, fibula, and humerus, but may also occur in other bony structures and cartilage tissue. This tumor was named after James Ewing, who in the 1920s described this small round blue cell tumor as being a separate entity from other histologically similar malignancies, such as lymphoma or neuroblastoma. It is the second most common malignant bone tumor after osteosarcoma, with the highest incidence in the second decade of life [1, 2]. The American Cancer Society estimates that 225 new cases are diagnosed annually in North America [1].

ES is the main member of a group of tumors known as Ewing's Sarcoma Family Tumors (ESFTs), which also contains peripheral primitive neuroectodermal tumors (pPNET). ES and pPNET are small round blue cell tumors; they were originally described as different entities; however, they are now recognized to represent ends of the morphologic spectrum of the ESFTs due to their close molecular relationship [3–7]. Some authors even assume pPNET and ES to be the same tumor with variable neural differentiation, a view that has been recently supported by immunohistochemical and cytogenetic findings [3]. The ESFT now includes osseous Ewing's sarcoma, EES, pPNET and Askin's tumor [7–9].

Ewing's sarcoma has two forms: the more common osseous Ewing's sarcoma (OES) and the relatively rare extraosseous Ewing's sarcoma (EES). EES has been reported in various tissues, including the chest wall, larynx, kidney, and esophagus. EES was first described by Tefft et al. in 1969, when they reported four patients with paravertebral soft tissue tumors histologically resembling ES [10]. Angervall and Enzinger in 1975 were the first to name this entity EES when they reviewed 39 patients with malignant soft tissue paravertebral tumors not arising from bone but having similar histologic characteristics to OES [11].

Spinal epidural EES in adults is a rare presentation among those locations where EES may occur. Here, we present an adult patient we recently treated, who represents only the 46th case of adult spinal epidural EES in the literature. Neurosurgeons should be aware of this rare clinical entity, which often presents with myelopathic and radicular symptoms associated with an epidural mass on imaging studies. Our review sheds light on the diagnosis, management, and prognosis of these cases.

## 2. Case Presentation

**Figure 1:**
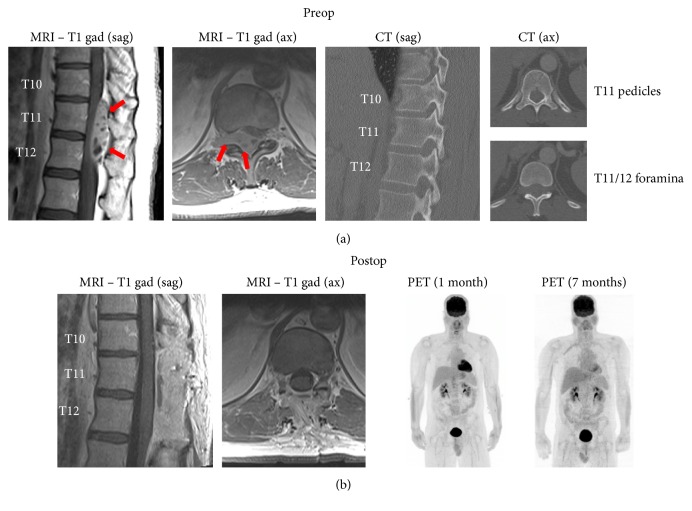
Radiographic findings. (a) Preoperative MRI indicates a heterogeneously enhancing epidural mass (arrows) at T10–12 extending from the spinal canal into the right T11-12 foramen. CT shows that the osseous elements are intact. (b) Postoperative imaging shows T10–12 laminectomies and gross total resection of the lesion. PET imaging 1 and 7 months after resection shows no abnormal FDG uptake. Sag: sagittal, ax: axial, and gad: gadolinium.

The patient is a 40-year-old male, previously healthy, who presented to the emergency department with several weeks of back pain and some urinary hesitancy lasting a few days. MRI of the thoracic spine indicated a heterogeneously enhancing extradural mass within the spinal canal at T10–T12, causing severe cord compression (Figure 1(a)). The mass was extended through the right neural foramina at T11-12 and T12-L1. CT did not suggest osseous involvement (Figure 1(a)). There were no other spinal lesions on MRI. CT of the chest, abdomen, and pelvis did not reveal any extraspinal sites suspicious for tumor growth. There were a number of somewhat enlarged periceliac lymph nodes of uncertain significance.

The patient underwent a T10–12 laminectomy for gross total resection of the tumor (Figure 1(b)), with preservation of motor and sensory function, resolution of urinary hesitancy, and significant improvement in the back pain. Resection of the foraminal component of the tumor required ligation and amputation of the right T11 nerve root.

**Figure 2:**
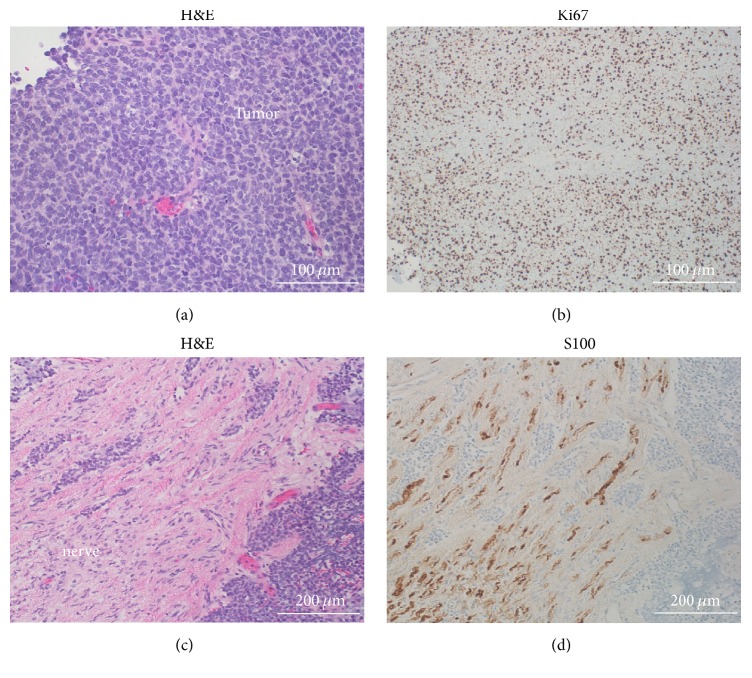
Histologic findings. (a) H&E stain within the tumor shows the small round blue cell appearance. (b) Ki67 immunostaining indicates a very high mitotic index (60–70%). (c) H&E stain demonstrates tumor invasion through the perineurium and into the right T11 nerve root. (d) Tumor invasion in the T11 nerve root is demonstrated by tumor cells interspersed within S100-positive Schwann cells. The tumor itself was S100-negative. H&E: hematoxylin & eosin.

Pathologic examination indicated a small round blue cell neoplasm (Figure 2(a)) composed of primitive densely packed cells with a very high mitotic index (60–70% of cells positive for Ki67) (Figure 2(b)). Molecular studies showed the EWSR1 rearrangement, confirming the diagnosis of Ewing's sarcoma. The tumor itself was negative for S100/chromogranin/synaptophysin and CD45/CD20, thus ruling out the small round blue cell tumors: pPNET and lymphoma, respectively. Microscopic analysis of the resected right T11 nerve root showed tumor invasion through the perineurium (Figures 2(c) and 2(d)).

Postoperative MRI and PET scan did not reveal residual or metastatic tumor (Figure 1(b)). The previously noted periceliac lymph nodes did not show increased FDG uptake on PET scan. Six weeks after surgery he started adjuvant chemotherapy consisting of ifosfamide (supplemented with mesna), cyclophosphamide, doxorubicin, and irinotecan. Eleven weeks after surgery he began adjuvant radiotherapy (45 Gy in 25 doses). He has tolerated all treatments well. He has no evidence of disease on repeat PET scan and MRI of the thoracic spine one year after surgery.

## 3. Discussion

### 3.1. Epidemiology

Spinal epidural EES in adults represents a very small fraction of spinal epidural masses and a rare presentation among those locations where EES may occur. We performed literature searches on PubMed to identify reports of spinal EES. We identified 119 cases of spinal EES in the literature from 1969 to 2015. In 43 of these cases the tumor was intradural, while it was localized to the epidural space in 76 cases. Of the epidural EES cases, 31 cases were pediatric patients and 45 were adults (Table 1). Treatment of these patients commonly required a combination of surgery, chemotherapy, and radiotherapy. The case we present here is the 77th reported case of spinal epidural EES and only the 46th case of adult epidural EES in the literature.

The review of the literature on combined pediatric and adult spinal EES/pPNET showed that the lumbar region is the most common site, followed by the thoracic and cervical spine, with the sacral region being the least common (5% of cases) [7, 12]. However, in our analysis of the 46 adult epidural EES/pPNET cases (including our case), we found that the most common site was the thoracic spine (17 cases), followed by lumbar (13 cases), cervical (13 cases), and sacral segments (3 cases).

Spinal EES in adults shows a predilection for males (61% of the cases), with a male : female ratio of 1.6 : 1, similar to that of OES [13, 14]. The average age at diagnosis was 29 years in contrast to 12 years for OES, and the oldest age reported was 65 years [12]. Interestingly, although it is stated that ES is rare in the Asian population [13, 14], we have found that half of the spinal EES patients in our study are Asians. However, no comprehensive epidemiological conclusions can be extracted due to the paucity of cases reported in the literature.

The mean diagnostic delay calculated from the previous cases is 4.5 months [7], which is explained by nonspecific symptoms at disease onset. The symptoms commonly include back and/or radicular pain in all patients, paresis in about 70%, sensory disturbances in 35%, and to lesser extent bladder and bowel dysfunction in about 12% of patients [7, 15]. One case presented with infection superimposed upon spinal epidural EES [16]. Distant metastases occurred in nearly 40% of the cases, either during or after diagnosis. Lung, spine, and brain were the most frequent sites of metastasis [7].

### 3.2. Histopathology

Ewing's sarcoma shows vague lobular proliferation of uniform small round blue cells with clear to lightly eosinophilic cytoplasm, evenly dispersed chromatin, and indistinct nucleoli. Peripheral PNET may specifically contain neuroblastic pseudorosettes termed Homer-Wright rosettes [3, 15, 17].

Immunohistochemical studies show that EES/PNETs strongly express cell surface glycoprotein CD99 (MIC2). This biomarker is considered one of the most accurate diagnostic tools and is positive in more than 90% of EES/pPNET cases. However, it is not exclusively specific for these tumors [18].

Approximately 25% of EES/pPNETs demonstrate aberrant expression of keratins, typically considered an epithelial marker. Expression of at least two different neuroglial antigens, such as neuron-specific enolase (NSE), protein S100, chromogranin, or synaptophysin, is required to distinguish pPNET from ES, with the former typically showing more neuronal differentiation [3, 4, 6, 15, 17, 19]. The tumor of the patient presented here was negative for S100, chromogranin, and synaptophysin, suggesting that the EES diagnosis was favored over pPNET.

At the genetic level, more than 90% of ES/pPNETs contain the same t(11;22)(q24;q12) translocation. Other translocations occur in 5–10% of cases [13]. The t(11;22)(q24;q12) translocation results in the formation of a chimeric gene (EWSR1-FLI1), which has been found to act as an oncogenic transcription factor in ES and pPNET [5, 6, 20]. This translocation can be detected by fluorescent in situ hybridization (FISH) in the nuclei of neoplastic cells. In its latest guidelines, ESMO (European Society for Medical Oncology) recommends that molecular studies be done to confirm the diagnosis of ESFTs through detection of this stereotypical translocation by FISH or RT-PCR [13].

### 3.3. Imaging Studies

Imaging studies are quintessential in such cases. MRI plays a prominent role in diagnosis, determination of the anatomic relationships with surrounding structures, and preoperative surgical planning. Commonly, EES/pPNET tumors have hypo- or isointense signal on T1-weighted imaging and a hyperintense signal on T2-weighted imaging and enhance heterogeneously. However, these MRI findings are nonspecific. In 15 case reports documenting MRI features of spinal epidural EES, the tumors were dumbbell-shaped and extended from the central canal toward widened foramina. In 3 cases, scalloping of bone was seen [20, 21].

Some reports suggest that the combination of FDG-PET with conventional imaging is a superior and valuable tool for disease staging and detecting metastases [22, 23].

In a recent study, O'Neill et al. proposed the concept of targeted imaging, using ^64^Cu-radiolabeled anti-CD99 antibody to detect these tumors and potential metastases. They found higher sensitivity with this approach as compared to FDG-PET in preclinical models [24].

### 3.4. Treatment and Prognosis

Surgical intervention is considered the primary and main approach in the management of these cases, particularly to relieve cord compression symptoms, as well as for cytoreductive purposes. Our analysis of previous cases showed that gross total resection (GTR) correlates with a much better outcome and decrease in recurrence rate than subtotal (partial) resection. Of the reported 46 cases in this paper, 45% had a subtotal resection (STR), while 55% of the cases had a GTR. Although partial resection has an increased risk of recurrence, complete resection is often precluded by tumor infiltration to the surrounding neural and paraspinal tissues.

Evidence from the literature strongly supports the use of local RT and systemic chemotherapy for treatment of EES/pPNET. Chemotherapy regimens for OES are often followed in adults with EES/pPNET. In the past, a traditional regimen was VACA (vincristine, actinomycin, cyclophosphamide, and/or doxorubicin). The addition of ifosfamide and/or etoposide to that regimen was the subject of many studies. In 1998, Ferrari et al. reported that ifosfamide/etoposide added to the induction, and maintenance phase of chemotherapy along with VACA resulted in a significantly better outcome in terms of histologic response and overall survival (OS) [25]. Other studies showed that adding ifosfamide and/or etoposide resulted in significant higher 5-year progression-free survival (PFS) and OS in nonmetastatic ESFTs. However, it did not improve outcomes in metastatic cases [15, 26].

Currently, the guidelines for treatment of ESFTs consider VAC/IE as the preferred first-line regimen for localized disease, concurrently with radiotherapy (45 Gy in 25 fractions). Regimens such as VAdriaC (vincristine, adriamycin, and cyclophosphamide) are used to treat metastatic disease [27]. Most of the recent adult spinal epidural EES/pPNET cases we reviewed followed such protocols postoperatively. We found that neoadjuvant chemotherapy was not used in any of these cases, despite its frequent use in the treatment of OES and other forms of EES. We postulate that neoadjuvant therapy may be of limited use in spinal epidural EES, due to the superior need for surgical decompression of the spinal cord.

Patients who underwent combined chemoradiotherapy after GTR or STR had better 1-year survival rates than patients treated with surgery, chemotherapy, or radiotherapy alone (88% versus 70%, resp.) [7]. In our study, 34 (74%) patients received combined chemoradiotherapy after GTR or STR; 2 (4%) cases underwent surgery only while chemotherapy and radiotherapy were given alone after surgery to 6 (13%) and 4 (9%) cases, respectively.

The prognosis of adult spinal epidural EES/pPNET is poor compared to OES. A study at Dana-Farber Cancer Center concluded that age plays an important prognostic factor, as survival rates are reduced in older adults. Also, primary extraosseous tumor and metastatic disease at diagnosis were adverse prognostic factors, even though both chemotherapy and radiotherapy were administered to patients with those three risk factors [28]. Another study reported that the 2-year survival rate in all spinal EES/pPNET cases was only 50% [7]. Furthermore, the 5-year survival rate in spinal epidural EES/pPNET is considered poor compared to other malignancies within the ESFT family. The 5-year survival rate of EES has been between 38% and 67%; however, the 5-year survival rate in spinal EES/pPNET ranged between 0 and 37.5% [20] (see also the follow-up and outcome data in Table 1).

## 4. Conclusions

Although primary spinal epidural EES/pPNET in adults is extremely rare, it should be considered in the differential diagnosis of patients with a history of nonspecific back pain and/or radicular pain, especially if accompanied by abnormal neurological examination and an epidural mass on MRI. The disease has an aggressive course, as evidenced by a high incidence of metastases and low survival rates reported in the literature. Early recognition of the disease entity and definitive management is essential. A multidisciplinary approach is the best strategy to manage epidural EES/pPNET, with surgical excision often being the initial intervention, due to neurological symptoms arising from spinal cord compression. Surgical resection should be followed by a combination of adjuvant chemotherapy and radiotherapy to improve overall outcome.

## Figures and Tables

**Table 1:** Cases of adult primary spinal epidural EES/PNET tumors in the literature.

Author	Year	Age (years)/sex (M or F)	Location/diagnosis	Treatment	Follow-up (months)	Outcome	CD99	t(11:22)	Country^*∗*^
Angervall and Enzinger [11]	1975	20/M	T2–T5/EES	STR/RT/CT	12	DOD	NA	NA	Sweden
Angervall and Enzinger [11]	1975	18/F	L5/EES	GTR/RT/CT	6	DOD	NA	NA	Sweden
Scheithauer and Egbert [29]	1978	18/M	L1/EES	GTR/RT/CT	16	NED	NA	NA	USA
Scheithauer and Egbert [29]	1978	27/F	T4–T6/EES	STR/RT/CT	132	NED	NA	NA	USA
Mahoney et al. [30]	1978	23/M	S1/EES	Biopsy/RT/CT	12	DOD	NA	NA	USA
Fink and Meriwether [31]	1979	19/M	L2-L3/EES	STR/RT/CT	12	NED	NA	NA	USA
N'Golet et al. [32]	1982	29/M	T1–T3/EES	GTR/RT/CT	6	NED	NA	NA	France
N'Golet et al. [32]	1982	47/F	L4/EES	GTR/RT/CT	4	DOD	NA	NA	France
Sharma et al. [33]	1986	18/M	T10/EES	STR/RT/CT	42	DOD	NA	NA	India
Liu et al. [34]	1987	26/F	L5-S1/PNET	STR/RT	6	NED	NA	NA	Taiwan
Christie et al. [35]	1997	36/F	L2-L3/EES	STR/RT	96	DOD	NA	NA	Australia
Dorfmüller et al. [3]	1999	18/M	L3-L4/PNET	GTR/RT/CT	23	NED	+	+	Austria
Kennedy et al. [36]	2000	24/M	C1–C5/EES	STR/RT/CT	13	NED	NA	NA	Ireland
Shin et al. [37]	2001	38/M	C5-C6/EES	STR/CT	17	NED	+	NA	South Korea
Shin et al. [37]	2001	22/F	C7-T1/EES	STR/CT	48	NED	+	NA	South Korea
Morandi et al. [38]	2001	22/F	T4-T5/EES	GTR/RT/CT	66	NED	+	NA	France
Morandi et al. [38]	2001	25/F	L1-S2/EES	STR/CT	7	DOD	+	NA	France
Mukhopadhyay et al. [15]	2001	29/F	C3–C5/EES	STR/RT/CT	30	NED	+	NA	India
Mukhopadhyay et al. [15]	2001	18/M	T8-T9/EES	STR/RT/CT	18	NED	+	NA	India
Mukhopadhyay et al. [15]	2001	22/M	L5-S1/EES	Biopsy/RT/CT	15	NED	+	NA	India
Mukhopadhyay et al. [15]	2001	31/M	L3-L4/EES	STR/RT/CT	32	NED	+	NA	India
Gandhi et al. [39]	2003	33/M	T5–T10/EES	GTR/RT/CT	3	NED	+	NA	Canada
Weber et al. [40]	2004	26/M	L1-L2/PNET	GTR/RT/CT	16	NED	+	NA	Switzerland
Koudelova et al. [41]	2006	28/F	L1-L2/PNET	STR/RT/CT	24	NED	NA	NA	Czech Republic
Isefuku et al. [5]	2006	20/M	L5-S1/EES	STR/CT	15	DOD	+	+	Japan
Ozturk et al. [8]	2007	18/M	C6-T1/EES	GTR/CT	13	NED	+	NA	Turkey
Lakhdar et al. [16]	2008	24/F	C6-C7/EES	GTR/CT/RT	NA	NA	NA	NA	Morocco
Bozkurt et al. [42]	2007	28/M	C3–C5/EES	GTR/RT/CT	18	NED	+	NA	Turkey
Feng et al. [43]	2008	24/M	T8–T10/PNET	GTR/RT	14	NED	NA	NA	China
Musahl et al. [44]	2008	27/M	S1-S2/PNET	GTR/RT/CT	24	NED	NA	NA	USA
Theeler et al. [9]	2009	28/F	T6/NS	GTR/CT	2	NED	+	+	USA
Kiatsoontorn et al. [45]	2009	25/M	T7/PNET	GTR/RT/CT	6	NED	+	NA	Japan
Jingyu et al. [19]	2009	58y/M	T4/PNET	GTR Only	25	NED	+	NA	China
Duan et al. [4]	2011	26/F	T4–T7/PNET	STR/RT/CT	3	NED	+	NA	China
Duan et al. [4]	2011	34/M	T12/PNET	STR Only	1	NED	+	NA	China
Yasuda et al. [20]	2011	37/F	T8-T9/EES	STR/RT/CT	22	DOD	+	+	Japan
Bostelmann et al. [46]	2011	29/M	C7/EES	STR/RT/CT	6	DOD	+	NA	Germany
Saeedinia et al. [7]	2012	44/F	S1–S3/NS	GTR/RT	9	NED	+	NA	Iran
Zhu et al. [21]	2012	46/M	C3–C6/EES	STR/RT/CT	12	DOD	+	NA	China
Zhu et al. [21]	2012	27/M	C1–C4/EES	GTR/RT/CT	10	NED	+	NA	China
Zhu et al. [21]	2012	27/M	C7/EES	GTR/RT/CT	24	NED	+	NA	China
Zhu et al. [21]	2012	24y/M	C5/EES	STR/RT/CT	7	DOD	+	NA	China
Kazanci et al. [12]	2015	34/F	T4–T6/EES	GTR/RT/CT	18	NED	+	NA	Turkey
Kazanci et al. [12]	2015	65/F	T7-T8/EES	GTR/RT/CT	14	NED	+	NA	Turkey
García-Moreno et al. [47]	2015	45/F	C6-T3/EES	STR/RT/CT	8	NED	+	+	Spain
Present case	2015	40/M	T10–T12/EES	GTR/RT/CT	12	NED	NA	+	USA

M: male. F: female. EES: extraskeletal Ewing's sarcoma. PNET: peripheral neuroectodermal tumor. GTR: gross total resection. STR: subtotal (partial) resection. RT: radiotherapy. CT: chemotherapy. NED: no evidence of disease. DOD: dead of disease. NS: not specified.

^*∗*^Country: the country where the cases were reported and studied.
